# Purification and Characterization of Transglutaminase Isolated from Sardine (*Sardina pilchardus*) Flesh Waste

**DOI:** 10.3390/polym17040510

**Published:** 2025-02-16

**Authors:** Imen Zaghbib, Johar Amin Ahmed Abdullah, Mnasser Hassouna, Alberto Romero

**Affiliations:** 1 Laboratory of Technological Innovation and Food Security LR22-AGR01, Higher School of Food Industries of Tunisia (ESIAT), University of Carthage, 58 Alain Savary Street, El Khadhra City, Tunis 1003, Tunisia; izaghbib@us.es (I.Z.); Mnasser.Hassouna@isbb.rnu.tn (M.H.); 2 Department of Chemical Engineering, Faculty of Chemistry, Universidad de Sevilla, 41012 Seville, Spain

**Keywords:** transglutaminase, sardine, enzyme purification, enzyme characterization, cross-linking, actomyosin

## Abstract

Transglutaminase (TGase) is an enzyme that catalyzes acyl transfer reactions by creating covalent cross-links between protein molecules and has been used to improve the physical and functional properties of protein-based foods. The objectives of this study were the extraction, purification, and biochemical characterization of TGase from sardine (*Sardina pilchardus*) flesh in order to provide a suitable TGase enzyme for food industry applications. The results showed a specific activity, yield, and purification fold of 357.14 U/mg protein, 36.74%, and 183.15, respectively. The enzyme exhibited maximal activity at 40 °C and pH 8.0, with a molecular weight of around 57 kDa. The effect of time on TGase thermal stability at 40 °C showed a gradual decrease in its catalytic activity during the incubation time until the enzyme was completely inactivated at 60 min. Additionally, the sardine TGase was found to be calcium-dependent. However, Mg^2+^ and Ba^2+^ ions were found to be effective in its activation to some extent and a total inhibition was shown by Zn^2+^ and Sr^2+^ ions. The TGase activity was affected markedly by NaCl and EDTA, and lost, respectively, about 80.7% and 36.49% from its activity by increasing the concentration (1.5 M NaCl and 20 mM EDTA). Based on the surface hydrophobicity and solubility results, the cross-linking of natural actomyosin mediated by TGase increased to a greater extent. The results revealed that sardine TGase possessed attractive qualities, making it a potential alternative to other TGase sources for food industry applications.

## 1. Introduction

Transglutaminase (TGase, EC 2.3.2.13) is an enzyme belonging to the class of transferases, which catalyzes the acyl transfer reaction between the γ-carboxamide groups (-(C=O)NH_2_) of the L- glutamine residues in peptide chains, acting as acyl donors, and the ε-amino groups (-NH_2_) of lysine residues, acting as acyl acceptors [1]. TGase can form ε-(γ-Glu)-Lys covalent bonds and, thus, create inter- or intramolecular cross-links leading to protein polymerization. This reaction can be used to enhance the texture, hydration properties, thermal stability, and other functional properties of certain foods, such as meat and dairy products, and particularly protein gel-based food systems [2]. Because of their distinctive qualities, TGases are better suited for particular applications and reaction conditions, promoting the cross-linking of proteins via enzymatic processes that offer advantages during food processing [3]. Binsi and Shamasundar [4] reported that the endogenous transglutaminase (TGase) enzyme plays a key role in the setting process, where fish muscle proteins are macerated with salt and incubated at temperatures below 40 °C. This process leads to the formation of covalent or non-covalent bonds. Gaspar and de Góes-Favoni [5] stated that the cross-links formed by TGase significantly modified the structure of the myosin-heavy chain, leading to a notable decrease in the α-helix content and an increase in the β-sheet structures. These structural changes facilitated the formation of high-molecular-weight polymers, thereby enhancing textural properties, including elasticity, stiffness, cohesion, and adhesiveness, resulting in strong gels with a compact and orderly structural conformation. In addition to its technological benefits, TGase has recently been used to enhance the nutritional quality of foods, thereby contributing to improved consumer health. Due to its ability to induce significant protein modifications, the addition of TGase to food products may help to regulate energy intake, reduce food allergies, and influence hormone growth [6].

It is well known that TGases are extensively found in nature (animals, plants, and microorganisms); however, commercial TGases are derived from limited sources. TGase was first discovered in guinea pig liver and recognized as an enzyme that catalyzes the incorporation of polyamines into the glutamine residues of proteins or peptides [7]. In microorganisms, TGase activity has been found in *Streptoverticillium sp.* and *Streptomyces sp.* [8]. In plants, TGase activity was identified in pea seedlings [9], the leaves of silver beet (*Beta vulgaris* L.) [10], soybean (*Glycine max*) leaves [11], and rosemary (*Rosmarinus officinalis* L.) [7]. Industrial applications of animal TGases were limited because of their scarce source, complicated isolation and purification processes, small yield, and expensive costs for large-scale production [4]. Among animal sources, the isolation and characterization of TGases from several fish species were developed by a few old studies [4,8,12,13,14,15], demonstrating the existence of a wide variation in their properties.

Since the development of food products and advancements in food processing are driving up the demand for enzymes, and in order to meet the global food shortages and the ever-challenging needs of a growing population, protein modification technology is gaining much interest [16]. Thus, finding more affordable and available sources of TGase is necessary, as its application in various food systems has been highly effective in enhancing textural properties [4].

The common sardine (*Sardina pilchardus*) is a small pelagic fish inhabiting the Mediterranean Sea and part of the eastern Atlantic [17]. Sardine is one of the most fished and processed species, with a global market size reaching 3.64 million tons in 2023, and is one of the main marine products traded internationally [18]. Globally, the Food and Agriculture Organization (FAO) estimated an annual discard in pelagic fisheries to be 2.2 million tons, which is a large quantity of fish being lost because of the large catch volumes [19]. Moreover, the expansion of the fisheries and aquaculture industry has resulted in the substantial generation of large amounts of by-products (between 20 and 80% of the remaining material) that are underused, wasted, or discarded [20]. The canning process of oily fish, such as sardine, sardine-type fish, and mackerel, gives rise to significant amounts of fish flesh residues containing important quantities of proteins [21]. In this context, implementing a waste management strategy that involves the reuse of these by-products into high-added-value products could be an effective way of valorization.

In recent years, sardine has been extensively studied as a source for various proteolytic enzymes, including trypsin and pepsin [20,21]. However, to date, the extraction and purification of sardine TGase have only been developed in two studies [4,13]. Binsi and Shamasundar [4] isolated and characterized transglutaminase from Indian oil sardine, which exhibited a specific activity of 66.35 units/mg, a molecular weight in the range of 73–95 kD, and was Ca^2+^ dependent. Batista et al. [13] carried out a preliminary characterization of sardine (*Sardina pilchardus*) transglutaminase in terms of optimal temperature, which was found to be around 35 °C, thermal stability in the range of 15–55 °C, as well as the influence of the ice storage of sardine on the stability of this enzyme where a slight decrease of TGase activity was recorded (about 50% of its initial activity after eight days in ice).

Despite all TGases sharing similar structural and functional characteristics, their molecular and immunological properties can vary, requiring different purification methods [4]. Therefore, the investigation of a standard, simple, and optimized sardine TGase extraction–purification protocol and a study of the enzyme’s biochemical characteristics are essential not only for updating the enzymology database but also for providing an appropriate enzyme for industry applications to meet the growing demands for enzymes.

In this context, the aim of the present study was to extract, purify, and investigate the enzymatic properties of the endogenous transglutaminase from sardine (*Sardina pilchardus*) flesh as a potential source of TGase for food industry use. TGase was characterized based on its molecular weight, optimal pH and temperature, thermal stability, enzyme concentration, and activity profile in the presence of activators and inhibitors. Additionally, the effectiveness of the purified sardine TGase in facilitating the setting and cross-linking of natural actomyosin was assessed.

## 2. Materials and Methods

### 2.1. Materials

Sardines (*Sardina pilchardus*) with an average length of 13–15 cm were obtained directly from a local Tunisian port. Samples were kept in ice and transported to the laboratory less than 12 h after catching, where they were hand-beheaded, eviscerated, filleted, washed, and minced.

All the chemicals and reagents used in this study were of analytical grade. Monodansylcadaverine (MDC) (C_17_H_25_N_3_O_2_S), N’N-dimethylated casein (DMC), bovine serum albumin (BSA), ethylenediaminetetraacetic acid (EDTA) (C_10_H_16_N_2_O_8_), dithiothreitol (DTT) (C_4_H_10_O_2_S_2_), ammonium sulfate ((NH_4_)_2_SO_4_), sodium chloride (NaCl), calcium chloride (CaCl_2_), barium chloride (BaCl_2_), magnesium chloride (MgCl_2_), zinc chloride (ZnCl_2_), strontium chloride (SrCl_2_), sodium dodecyl sulfate (SDS), TEMED (*N*,*N*,*N*′,*N*′-tetramethylene diamide), acrylamide, bis-acrylamide (*N*,*N*′-methylene-bisacrylamide), β-mercaptoethanol, Trizma base (tris(hydroxymethyl) aminomethane), Coomassie Brilliant Blue R-250, and standard protein markers were purchased from Sigma-Aldrich Inc. (St. Louis, MO, USA).

### 2.2. Transglutaminase Extraction and Purification

#### 2.2.1. Preparation of the Crude Transglutaminase Extract

**Figure 1:**
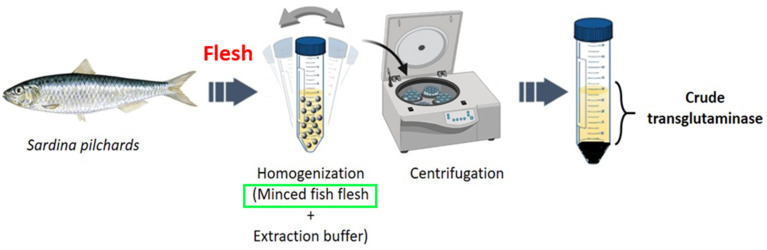
Extraction steps of crude transglutaminase from sardine (*Sardina pilchardus*) flesh.

The crude enzyme extract was prepared according to the method of Binsi and Shamasundar [4] with slight modifications (Figure 1). Fish samples were homogenized with the extraction buffer (10 mM NaCl, 5 mM EDTA, 2 mM β-mercaptoethanol, 10 mM Tris–HCl, pH 7.5) in a ratio of 1:4 (weight: volume) at 9000 rpm for 30 min using an Ultra Turrax homogenizer (Ultra-Turrax, T 25, Janke & Kunkel GMBH & Co., KG Staufen, Germany). The homogenate was centrifuged at 9000 g/30 min at 4 °C using a refrigerated centrifuge (Kendro Laboratory Products, Newton, CT, USA). The sediment was neglected, and the supernatant was used to extract the crude TGase.

#### 2.2.2. Purification of Sardine Transglutaminase

**Figure 2:**
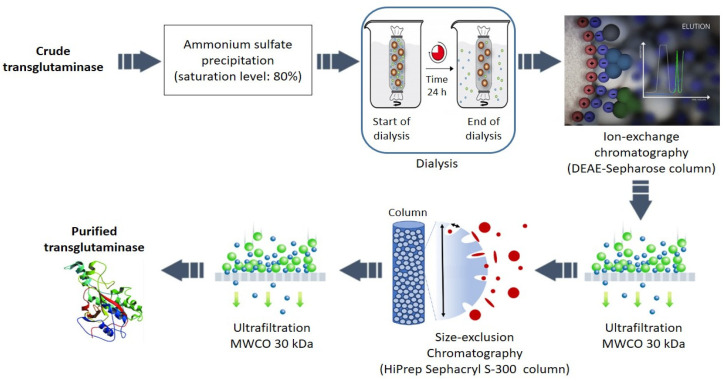
Purification process of the transglutaminase enzyme.

The crude TGase extract was purified as described by Tsukamasa et al. [22] with slight modifications. The purification protocol, as shown in Figure 2, essentially involves:

(i). Ammonium sulfate precipitation: The crude enzyme was precipitated using ammonium sulfate at a saturation level of 80%. This was followed by the dialysis of the enzyme against a large volume of the same extraction buffer for 24 h, with the buffer replaced every 6 h to remove the ammonium sulfate salts.

(ii). Ion-exchange chromatography: The dialyzed fraction was applied into a DEAE-Sepharose column (40 × 12.5 mm), equilibrated with the extraction buffer (10 mM NaCl, 5 mM EDTA, 2 mM β-mercaptoethanol, 10 mM Tris–HCl, pH 7.5), to remove the unbound proteins. The target components (proteins) were eluted with a linear gradient of 0–1 M NaCl, prepared in the same buffer at a flow rate of 60 mL/h. Fractions (2 mL) were collected using a fraction collector (Bio-rad, Richmond, CA, USA). Their protein concentration at 280 nm was determined, and the TGase activity was assayed. Fractions with TGase activity were pooled and concentrated using a Millipore ultrafiltration membrane (from 15 mL to 1.5 mL) with the molecular weight cut-off (MWCO) of 30 kDa (Amicon Ultra-15 centrifugal filter, MilliporeSigma, Austin, USA).

(iii). Size exclusion chromatography: 5 mL of the concentrated TGase obtained from the DEAE-Sepharose column were further purified by application to a HiPrep Sephacryl S-300 column (1.6 × 60 cm), and equilibrated with the same buffer (10 mM NaCl, 5 mM EDTA, 2 mM β-mercaptoethanol, 10 mM Tris–HCl, pH 7.5) at a constant flow rate of 60 mL/h. Fractions of 2 mL were collected, their protein concentration at 280 nm was recorded, and the TGase activity was assayed. The rich fractions of the TGase activity obtained were pooled and concentrated using a Millipore ultrafiltration membrane (from 15 mL to 1.5 mL) with the molecular weight cut-off (MWCO) of 30 kDa (Amicon Ultra-15 centrifugal filter, Millipore, USA). The obtained fraction was considered as a purified enzyme (purified TGase). All the purification steps were conducted at 4 °C.

### 2.3. Characterization of Sardine Transglutaminase

#### 2.3.1. Transglutaminase Activity Assay

The TGase activity was determined regarding the incorporation of MDC into DMC following the procedure described by Worratao and Yongsawatdigul [12]. Briefly, 20 µL of sardine TGase was added to the reaction mixture comprised of 1.25 mg/mL DMC, 18.75 µM MDC, 3.75 mM DTT, 6.25 mM CaCl_2_, and 62.5 mM Tris-HCl (pH 7.5), and incubated at 37 °C for 10 min. The catalytic reaction was then stopped by adding the EDTA solution at a final concentration of 20 mM. The fluorescence intensity was measured with a spectrofluorophotometer (LS50B, PerkinElmer, Waltham, USA) at excitation and emission wavelengths of 350 and 480 nm, respectively. One unit (U) of enzyme activity was defined as the amount of TGase that catalyzes the incorporation of 1 nmol of MDC into DMC for 1 min at 37 °C. The specific activity was expressed as enzyme units/mg protein.

#### 2.3.2. Purification Fold and Yield of Transglutaminase

The purification fold and yield were determined at different steps of purification, according to the Formulas (1) and (2), as given below [4]:

Purification fold = Specific activity at a particular step/Specific activity of the crude enzyme(1)

Yield of extracted enzyme (%) = (Total activity at a particular step/Total activity of crude enzyme) × 100(2)

#### 2.3.3. Protein Content

The protein concentration was determined according to Bradford’s method [23] using a UV 1201-vis spectrophotometer (Shimadzu, Tokyo, Japan) at a wavelength of 595 nm. Bovine serum albumin (BSA) was used as the standard in the range of 2.5–30 μg/mL.

#### 2.3.4. SDS–PAGE Electrophoresis and Molecular Weight

The purity of TGase was analyzed using sodium dodecyl sulphate–polyacrylamide gel electrophoresis (SDS-PAGE) as described by Laemmli [24] with slight modifications. Appropriate volumes of each protein fraction at a concentration of 30 µg/mL were mixed with a SDS-PAGE loading buffer (62.5 mM β-mercaptoethanol, 1% bromophenol, 10% SDS, 20% glycerol, 0.5 M Tris-HCl, pH 6.8, distilled water) at a 4:1 ratio (v:v), and then heated at 100 °C for 5 min. Next, samples from each purification step were applied to the gel, made of 5% acrylamide stacking gel and 10% acrylamide separating gel. Protein markers (5 µL) containing 9 protein standards with molecular weights ranging from 15 to 250 kDa were also loaded. Electrophoresis was performed at 30 mA and 200 V for 40 min using an SDS-PAGE Protean II XL (Bio-Rad Laboratories, Inc., Richmond, CA, USA) until the tracking dye reached the bottom of the gel. After separation, the gels were stained using 0.5% Coomassie Brilliant Blue R-250 solution for 15 min. The gels were de-stained using a methanol:acetic acid de-staining solution (3:1, v:v). The gels were scanned using the Image Lab Digi-Doc-IT (UVP, Upland, Ca). The molecular weight of sardine TGase was then determined using standard curves constructed by plotting the logarithm of the MW as a linear function of the relative migration distance (Rf).

#### 2.3.5. Optimal pH

Determination of the optimal pH was conducted by preparing the reaction mixtures with various buffers at different pH values (pH 4–6.5 using 100 mM acetate buffer, pH 7–7.5 using 50 mM Tris-HCl, and pH 8–9 using 50 mM borate buffer). In order to define the TGase optimal pH, the relative TGase activity (%) was calculated as the percentage of the remaining activity in the presence of the reaction mixture using the highest TGase activity obtained as 100%.

#### 2.3.6. Optimal Temperature

The influence of temperature on the sardine TGase activity was determined at various temperatures. Tubes containing the reaction mixture and the extracted enzyme were incubated at 20, 25, 30, 35, 40, 45, 50, 55, 60, 65, and 70 °C for 10 min. In order to define the TGase optimal temperature, the relative TGase activity (%) was calculated using the highest TGase activity obtained as 100%.

#### 2.3.7. Thermal Stability

The thermal stability of TGase was conducted by incubating the purified enzyme at the optimal temperature for 0, 10, 20, 30, 40, 50, and 60 min, followed by rapid cooling to 37 °C, and analyzing immediately for the enzyme activity measurement. The TGase activity was estimated as a relative activity, representing the percentage of remaining activity after incubation at different times. For the calculations, the relative activity without incubation (0 min) was considered 100%.

#### 2.3.8. Effect of Enzyme Concentration on Transglutaminase Activity

The effect of the enzyme concentration on the transglutaminase activity was determined by testing different concentrations (0 U/mg, 7.14 U/mg, 14.28 U/mg, 21.43 U/mg, 28.56 U/mg, and 35.7 U/mg) of pure TGase and incubating at 37 °C for 10 min. For the calculations, the relative activity (%) was calculated using the highest TGase activity obtained as 100%.

#### 2.3.9. Effect of Salts, Chelating Agents, and Metal Ions on Transglutaminase Activity

The effects of various NaCl concentrations (0, 0.3, 0.6, 0.9, 1.2, and 1.5 M); CaCl_2_ (0, 2, 4, 6, 8, and 10 mM), used as an activator; and EDTA (0, 5, 10, 15, and 20 mM), used as an inhibitor of TGase activity were tested. Tubes containing the reaction mixture and the extracted enzyme were incubated at 37 °C for 10 min. The relative TGase activity (%) was calculated using the highest TGase activity obtained as 100%. The effect of metal ions on TGase activity was conducted by replacing CaCl_2_ with SrCl_2_, MgCl_2_, and BaCl_2_ at 10 mM in the reaction mixture. The effect of ZnCl_2_ was studied at 10 mM in the presence of 1.25 mM CaCl_2_. Samples were then incubated at 37 °C for 10 min. For the calculations, the highest relative activity of CaCl_2_ was taken as 100%.

### 2.4. Effect of Added Sardine Transglutaminase on the Natural Actomyosin (NAM) Cross-Linking

#### 2.4.1. Incubation of NAM with TGase

Sardine NAM solutions were prepared according to Hemung et al. [25]. For cross-linking, the NAM solutions were incubated at 25 °C/4 h and 40 °C/2 h with TGase in the reaction mixture containing 3 mg/mL NAM in 0.6 M NaCl, 5 mM CaCl_2_, 5 mM DTT, 20 mM Tris–HCl (pH 7.5), and 3 U TGase/mL. For the control, TGase was replaced with distilled water.

#### 2.4.2. Protein Solubility

The solubility of protein samples was measured following the method of Wang et al. [26]. NAM solutions were centrifuged at 1000× *g* for 20 min at 4 °C, and the protein content of the clear supernatant was determined according to Bradford’s assay [23] using bovine serum albumin (BSA) as a reference protein. The solubility was expressed as follows:

Solubility (%) = (C_S_/C_0_) × 100 (3)

where C_S_ represents the protein concentration after centrifugation (mg/mL); C_0_ represents the protein concentration before centrifugation (mg/mL).

#### 2.4.3. Surface Hydrophobicity

The surface hydrophobicity of the NAM samples was determined using the 8-anilino-1-naphthalenesulfonic acids (ANS) method as described by Benjakul et al. [27] with slight modifications. NAM was prepared in a 10 mM phosphate buffer (pH 6.0) containing 0.6 M NaCl to obtain a concentration of 1 mg/mL. The diluted protein (2 mL) was added with 10 µL of 8 mM ANS in a 0.1 M phosphate buffer of pH 7.0. The fluorescence intensity of the ANS–protein conjugates was measured using a spectrofluorometer (RF-1501 Shimadzu, Kyoto, Japan) at excitation and emission wavelengths of 374 nm and 485 nm, respectively. For the control, NAM without TGase was used. The protein hydrophobicity was calculated from the initial slopes of plots of relative fluorescence intensity vs. protein concentration (%, *w*/*v*) using a linear regression analysis. The initial slope was referred to as S_0_ANS.

### 2.5. Statistical Analysis

Data were subjected to the Analysis of Variance (ANOVA) using the Statistical Analyses Software (SAS, Version 9.1, Cary, NC, USA). A comparison among means was evaluated by performing Tukey’s test at the 5% significance level. The results were reported as the mean ± SD of the triplicate observations.

## 3. Results and Discussion

### 3.1. Transglutaminase Extraction and Purification

Purification is the process of separating specific enzymes from a crude cell extract that contains other undesirable elements in order to maximize the desired specific activity and the recovery of the initial activity [28]. After each purification step, the improvement in protein content, total and specific activity, purification fold, and yield were summarized in a purification scheme (Table 1). In the first stage, the crude TGase extract was subjected to ammonium sulfate precipitation at a saturation level of 80%. This step gave a specific activity, purification fold, and yield after ammonium sulfate precipitation of 0.68 U/mg protein, 0.35, and 98.11%, respectively. According to Westphal and van Berkel [29], the specific activity is a good indication of the enzyme preparation’s purity and quality; however, the purification fold provides an insight into the efficiency of each step.

**Figure 3:**
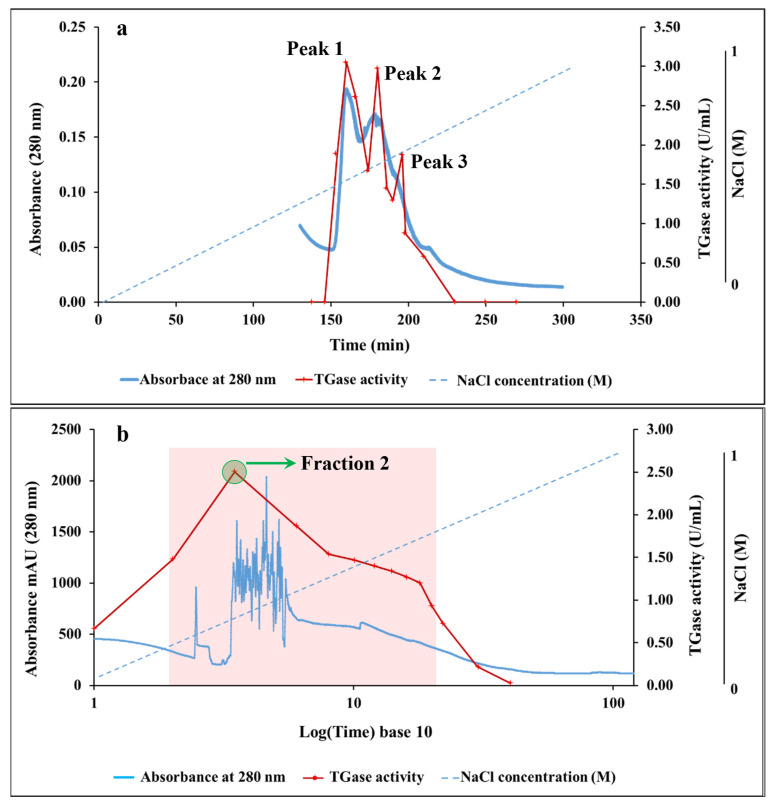
Chromatographic profiles at different purification steps of sardine TGase. (**a**) Elution from the DEAE-Sepharose column showing three TGase activity peaks (Peak 1, Peak 2, Peak 3). (**b**) Elution from the HiPrep Sephacryl S-300 column showing a single peak with maximum TGase activity at Fraction 2 (highlighted). Fractions 1 to 10 were pooled, concentrated, and considered as the purified enzyme.

The ammonium sulfate precipitate was subjected to DEAE-Sepharose column anion-exchange chromatography, and the elution profile is summarized in Figure 3a. Before introducing the dialyzed TGase extract of sardine muscle, the DEAE-Sepharose column was washed extensively with an extraction buffer until the absorbance at 280 nm decreased to a negligible level. A NaCl gradient elution (0–1 M) was used to elute the samples from the column. During this purification step, TGase was eluted between 0.05 and 0.53 M NaCl as three peaks with a yield, purification fold, and specific activity of 87.23%, 83.15, and 162.16 U/mg protein, respectively (Figure 3a and Table 1). The concentration of NaCl required to elute the TGase enzyme from the human epidermal was 0.2 M, rosemary (*Rosmarinus officinalis* L.) was between 0.2 and 0.3 M, Antarctic krill (*Euphausia superba*) was between 0.13 and 0.24 M, the dorsal muscle of carp, rainbow trout, and Atka mackerel was 0.3 M, while squids, scallop, and oyster TGase eluted at 0.16, 0.12, and 0.15 M, respectively [7,8]. The principle of elution using a linear salt gradient is to decrease the net charge of the proteins and obtain a high degree of protein fractionation. By increasing the salt concentration, the interaction between the resin and the enzymes is reduced. The molecules with the weakest ionic interactions start to elute from the column first. Molecules with a stronger ionic interaction require a higher salt concentration and elute later in the gradient [30].

Ion exchange chromatography (IEC) ranks among the most commonly utilized techniques for purifying enzymes. IEC is an effective purification method because: (i) IEC has a high capacity for binding, which enables proteins to be eluted in a concentrated form; and (ii) the selection of appropriate elution conditions leads to the separating of the bound proteins at a high resolution. IEC was a key step in enzyme purification for many years, both at a laboratory scale and at an industrial scale [29].

The final step of purification was achieved by collecting the enzyme fractions from the previous step, which were pooled and concentrated from 15 mL to 1.5 mL using an ultrafiltration membrane. The concentrated fraction was then loaded on the HiPrep Sephacryl S-300 chromatography column for further purification, and the chromatogram obtained is shown in Figure 3b. TGase was eluted as a single distinct peak, with the protein absorbance (A280) and TGase activity reaching a maximum at Fraction 2, as highlighted in the figure. Fractions 1 to 10, which demonstrated the highest TGase activity, were collected, pooled, and concentrated. These fractions were considered the purified enzyme. The sharpness of the single peak observed in the chromatogram indicates the effective separation and high purity of the TGase enzyme, achieved through the size-exclusion chromatography step. The yield, purification fold, and specific activity at this step were 36.74%, 183.15, and 357.14 U/mg protein, respectively (Figure 3b and Table 1).

According to Westphal and van Berkel [29], size exclusion chromatography (SEC), also referred to as gel filtration, is a suitable step for enzyme purification. SEC is very useful for obtaining information about the molecular weight of the native protein and its possible subunit composition.

In this study, the final yield and specific activity of the purified sardine TGase were considerably higher compared with the other marine fish species reported in previous studies (Table 2). Therefore, our extraction and purification method may be more suitable than those previously reported for industrial-scale TGase production.

### 3.2. Transglutaminase Characterization

#### 3.2.1. Purity and Molecular Weight

**Figure 4:**
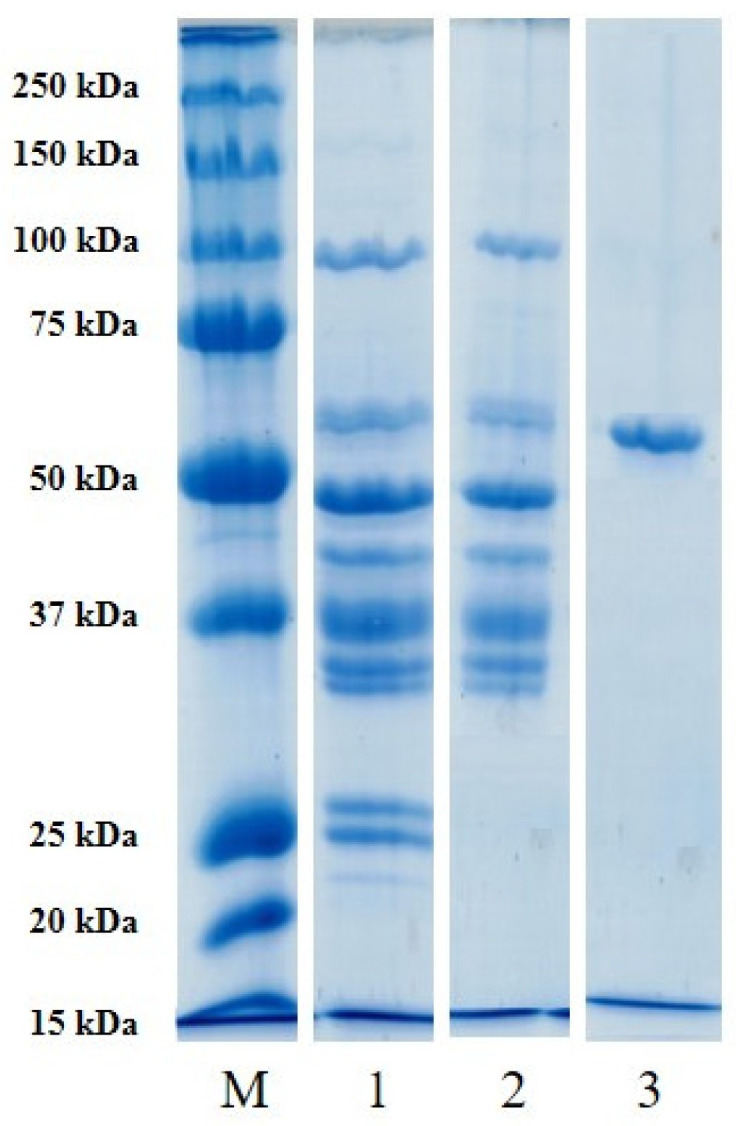
SDS-PAGE of samples obtained from each purification step. **M** standard markers; **1** ammonium sulfate fraction at 80% saturation; **2** Collected fractions after DEAE-Sepharose chromatography; **3** Collected fractions after HiPrep Sephacryl S-300 chromatography (purified TGase).

The purity and molecular weight of the extracted enzyme and the efficiency of the purification steps used were confirmed using electrophoresis SDS-PAGE (Figure 4). The SDS-PAGE pattern revealed a single major bond, free of detectable contaminants, at the final purification step, which indicated the purity of the enzyme. This also showed the efficiency of the protocol and the conditions used to extract and purify this enzyme. The molecular weight of pure sardine TGase, as derived from the standard curve, was estimated to be around 57 kD. The molecular weight of TGase obtained in this research was lower than that of other tissue TGases, including ordinary carp, pollock scallop, rainbow trout, and Atka mackerel, which were in the range of 80–100 kD, and from bigeye snapper, Indian oil sardine, tilapia (Oreochromis mossambicus), and common carp, which were in the range of 73–95 kD [4]. According to the study of Laksono et al. [31], it was seen that the molecular size of TGase from daggertooth pike conger fish (Muraenesox cinerus) meat was 80 kDa.

#### 3.2.2. Optimal pH

**Figure 5:**
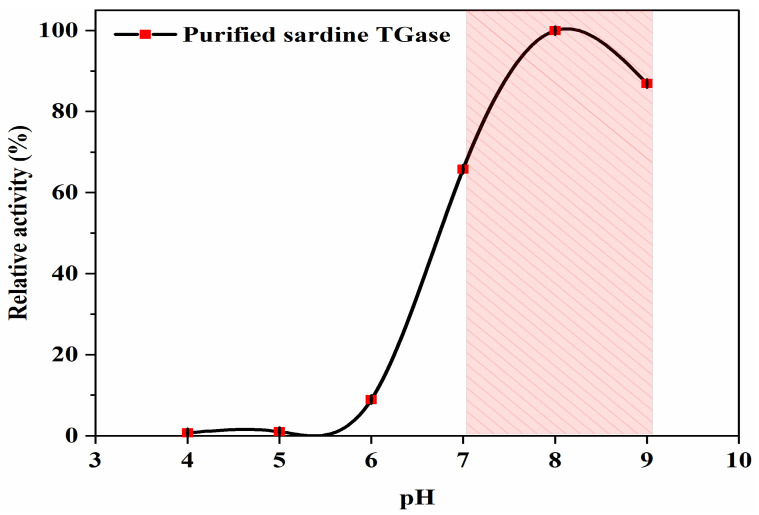
pH profile of the purified sardine TGase. The protein concentration used was 0.14 mg/mL. Bars represent the standard deviation (n = 3).

Because enzymes have several ionizable groups, they are active in a limited pH range. Changes in pH can affect the active site, the enzyme conformation, and essentially the catalytic reaction. Sardine TGase activity was determined under different pH conditions (from pH 4.0 to 9.0). As shown in Figure 5, the highest TGase activity was found over the pH range of 7.0 and 9.0, with an optimum recorded at pH 8.0. However, the enzyme seemed inactive in the acidic region (pH between 4.0 and 6.0), which is remarkable for the loss of total activity. The optimal pH was similar to those reported for Antarctic krill, scallops, carp, and Japanese oyster TGases (optimal pH 8.0) [14,32,33,34]. TGases from other fish species showed an optimal pH of 9.0 for walleye pollock liver and between 8.5 and 9.0 for threadfin bream liver [8]. pH stability is related to the structure of the enzyme, where amino acids with electrically charged side chains occur on the enzyme surface. When the pH value of the reaction medium changes, modifications in the enzyme structure are established due to the change in the charge distribution produced by the dissociation and association of the side chains. The disruption of the electrostatic interactions causes structural disturbances and conformational changes, leading to a loss of enzyme activity, which indicates denaturation due to extreme pH conditions [35].

#### 3.2.3. Optimal Temperature

**Figure 6:**
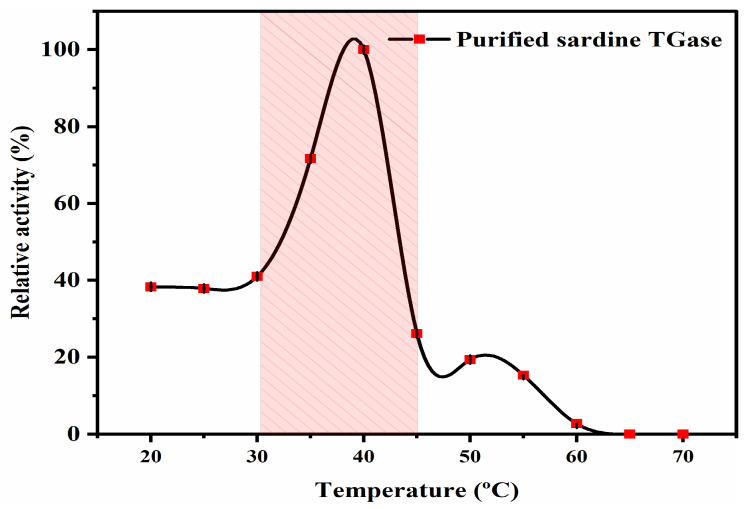
Temperature profile of the purified sardine TGase. The protein concentration used was 0.14 mg/mL. Bars represent the standard deviation (n = 3).

The TGase activity profile was obtained in the 20–70 °C temperature range, and the results were depicted in Figure 6. It was noted that the enzyme activity increased with the temperature to reach its maximum at 40 °C. This finding suggested that sardine TGase starts to unfold at relatively high temperatures. With increasing temperatures that were outside the optimal temperature range, TGase catalytic activity declined gradually from 40 to 70 °C until the enzyme completely lost its effectiveness. This observation indicated an unfolding transition and changes in the TGase molecular structure, and it could be concluded that sardine TGase is a heat-labile enzyme. This is in agreement with the study conducted by Binsi and Shamasundar [4], who indicated that TGase from oil sardine and common carp exhibited optimum activity at 37 °C, and were less stable at higher temperatures. A similar result was also found in the TGase extracted from daggertooth pike conger fish meat, which had an optimal temperature ranging from 40 to 50 °C [31]. Moreover, Klesk et al. [36] found that the “setting” temperature of surimi prepared from tilapia was around 40 °C. According to Hemung and Yongstwadigul [32], and Binsi and Shamasundar [4], the differences in the optimal temperatures for TGase activity may be related to several factors: (i) the habitat temperature of fish species; (ii) the extent of enzyme purity’ and (iii) the temperature stability of fish meat.

#### 3.2.4. Thermal Stability

**Figure 7:**
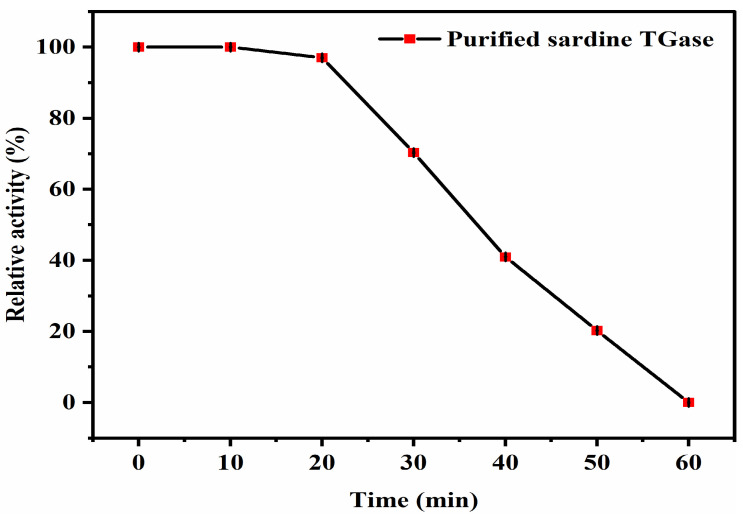
Thermal stability profile of the purified sardine TGase at 40 °C and different incubation times. The protein concentration used was 0.14 mg/mL. Bars represent the standard deviation (n = 3).

To gain more insight into the purified TGase thermal robustness, a thermal stability analysis was performed at the optimal temperature (40 °C) over an incubation period of 60 min by checking the activity at different time points (Figure 7). It can be seen that the enzyme retained its total activity (100%) between 0 and 20 min. After that, the activity decreased gradually during the incubation time and presented around 70.29% of its initial activity at 30 min. After this period, the TGase activity continued decreasing until it reached approximately 20.23% of its initial value after 50 min of incubation at 40 °C. This change in activity is more accentuated after 60 min of incubation, a time in which the enzyme was completely inactivated. This result indicated a significantly high loss of enzymatic activity after one hour of incubation in the reaction mixture at 40 °C. Furthermore, it suggested that sardine TGase was relatively stable at 40 °C within a short reaction duration and exhibited high time-dependence stability.

#### 3.2.5. Effect of Enzyme Concentration on Transglutaminase Activity

**Figure 8:**
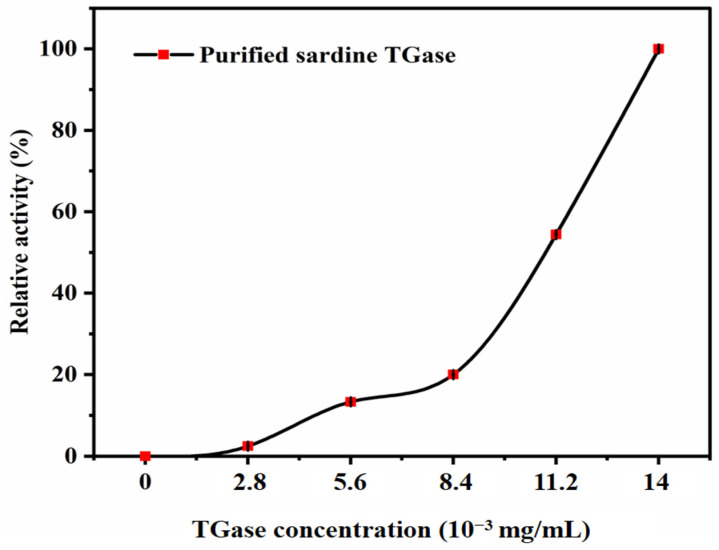
Effect of enzyme concentrations (mg/mL) on the purified sardine TGase activity. The protein concentration used was 0.14 mg/mL. Bars represent the standard deviation (n = 3).

The concentration of the enzyme is important in chemical reactions as it is needed to react with the substrate. The results of sardine TGase activity as a function of TGase volume (µL) are shown in Figure 8. It was noted that the rate of the TGase-catalyzed reaction was directly dependent on the enzyme concentration. In fact, with the increase in the enzyme concentration, the active sites’ effectiveness also increased; thus, TGase showed the highest activities (100%) at 14 × 10^−3^ mg/mL. The standard transglutaminase activity assay protocol adopted in this work [12] added 20 µL TGase (corresponding to 2.8 ×10^−3^ mg/mL) to the reaction mixture. However, the present study revealed that a higher enzyme concentration (≥14×10^−3^ mg/mL) is required for maximum TGase activity. This is because, with a higher amount of enzyme, the substrate had more active sites to which it could bind. Thus, more enzyme–substrate complexes were formed, leading to an increase in the enzyme activity. Figure 8 does not show that the enzyme activity leveled off (plateaued). Therefore, sufficient substrate molecules are available to react with the extra enzyme.

It is important to note, however, that enzyme activity is not solely dependent on the enzyme concentration but also on the substrate concentration, as per classical enzyme kinetics models such as the Michaelis–Menten equation. In the present study, the substrate concentration was kept constant. Figure 8 does not show that the enzyme activity leveled off (plateaued), suggesting that sufficient substrate molecules were available to react with the extra enzyme.

This approach provides useful information regarding the enzyme efficiency at different volumes, i.e., concentrations, but the results must be interpreted within the limitation that the substrate concentration was not varied. To obtain a more comprehensive understanding of the kinetics of sardine TGase, future studies should incorporate varying substrate concentrations, which would allow for the determination of key kinetic parameters, such as the Michaelis constant (K_m_) and maximum reaction velocity (V_max_). This would provide a deeper insight into the enzyme’s catalytic efficiency and potential application under industrial processing conditions.

#### 3.2.6. Effect of Chemical Additives on Enzyme Activity

Enzymes have unique forms and characteristics. The complexity in their molecular structure may cause impediments to their use. Thus, in order to maintain the required stability and activity of the enzyme, external factors, such as the presence of chemical compounds that can activate or inhibit their functions, must be taken into account [28].

##### Effect of Calcium Chloride (CaCl_2_)

**Figure 9:**
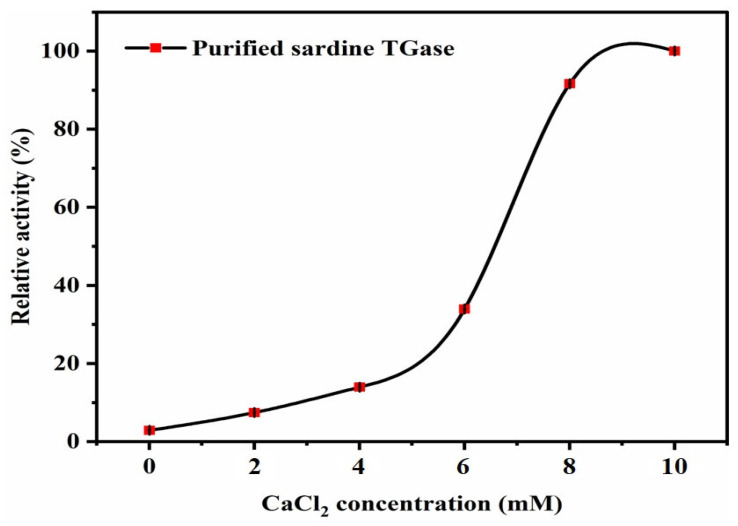
Effect of CaCl_2_ concentrations on the purified sardine TGase activity. The protein concentration used was 0.14 mg/mL. Bars represent the standard deviation (n = 3).

The effects of Ca^2+^ at the concentrations of 0, 2, 4, 6, 8, and 10 mM are presented in Figure 9, which shows that sardine TGase is absolutely dependent on calcium ions. In the absence of Ca^2+^, there was no activity observed. Thereafter, relative activity increased with Ca^2+^ concentration and reached a maximum at 10 mM. The present study revealed that the optimal Ca^2+^ concentration required for maximum TGase activity was higher than that specified in the standard transglutaminase activity assay protocol, as reported by Worratao and Yongsawatdigul [12] (a concentration of 6.25 mM CaCl_2_ was added to the reaction mixture of TGase). Moreover, determining the ideal Ca^2+^ concentration proved to be challenging due to the fact that the relative activity reached its peak at 10 mM and could either decrease or increase further beyond this concentration. A previous study on purified Antarctic krill TGase showed the same behavior towards calcium ions with an optimal Ca^2+^ concentration of 10 mM and, thereafter, declining until a concentration of 50 mM [8]. However, TGases from threadfin bream (*Nemipterus* sp.) liver showed the highest activities at Ca^2+^ concentrations less than 1 mM [32]. It was also reported that the maximum activity of TGase from rosemary (*Rosmarinus officinalis* L.) leaves was recorded at 2 mM Ca^2+^ [7]. Alhasani and Al-Younis [2] noted that TGase purified from chard was Ca^2+^-independent since there was no effect of Ca^2+^ ions on the enzyme activity. From these findings, it could be confirmed that TGases from various sources and species require different Ca^2+^ concentrations for full activation. The significance of Ca^2+^ ions in TGase cross-linking reactions has been widely acknowledged; however, there remains a limited understanding regarding the structural and functional reasons behind the necessity of Ca^2+^ ions. Several previous studies have tried to explain this observation. Calcium ions are called activators since they enable the TGase to bind better with its substrates by inducing conformational changes in the enzyme active site and causing an increase in the enzyme reaction speed. Cysteine located at the active site will be better exposed and consequently binds with the acyl donor, forming an acyl–enzyme intermediate [8,12,31]. This property proves to be highly advantageous in the food industry when it comes to modifying many proteins (such as myosin, soybean, and milk casein), which are highly sensitive and tend to precipitate easily in the presence of Ca^2+^ ions [7].

##### Effect of Sodium Chloride (NaCl)

**Figure 10:**
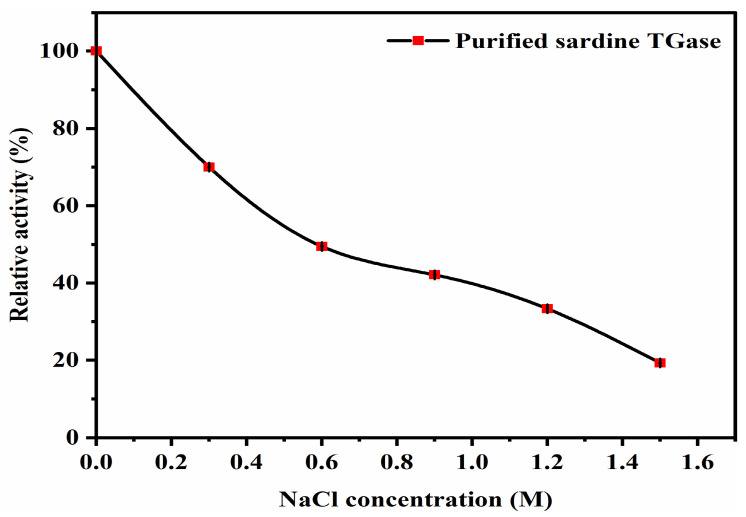
Effect of NaCl concentrations on the purified sardine TGase activity. The protein concentration used was 0.14 mg/mL. Bars represent the standard deviation (n = 3).

The relative activity of TGase was studied in the presence of various sodium chloride concentrations (0.3–1.5 M) (Figure 10). The sardine TGase showed high activity at 0–0.3 M NaCl, which is the common salt content (approximately 1.8% NaCl) typically employed during fish protein gel preparation to solubilize muscle proteins [32]. Thereafter, higher NaCl concentrations significantly affected the TGase activity, which reduced gradually until it reached a relative activity of 50% at 0.6 M NaCl. Above 1.5 M NaCl, the relative activity recorded was 19.3%. The decrease in the TGase activity could be due to the conformational changes induced in the enzyme active site by NaCl addition. This result was in agreement with the findings of Hemung and Yongsawatdigul [32], who noted that partially purified TGase from threadfin bream liver exhibited a high activity at 0.3 M NaCl and then slightly reduced, until reaching a remaining activity of 75% at 1.2 M. Worratao and Yongsawatdigul [12] stated that the TGase activity of the freshwater fish “tropical tilapia” was markedly inhibited by NaCl above 0.5 M. Previous observations made with TGase derived from Antarctic krill showed increased activity by adding 1.8 mM NaCl (relative TGase activity of 139.29 ± 0.74%) [8]. These findings indicated a strong correlation between the environmental habitat of marine species and the optimal conditions for their enzyme activity. According to Hemung and Yongsawatdigul [32], the enhancing effect of NaCl was found especially in marine invertebrates because when their muscles are injured, TGase is released into the extracellular space to participate in the healing of the wound. As a result, during its catalytic reaction, the enzyme is exposed to a high concentration of NaCl in its surrounding environment. The result of this study confirmed that sardine TGase could be applied in the food industry to produce food-based proteins containing NaCl up to 1.5 M without a total loss in activity.

##### Effect of Ethylenediaminetetraacetic Acid (EDTA)

**Figure 11:**
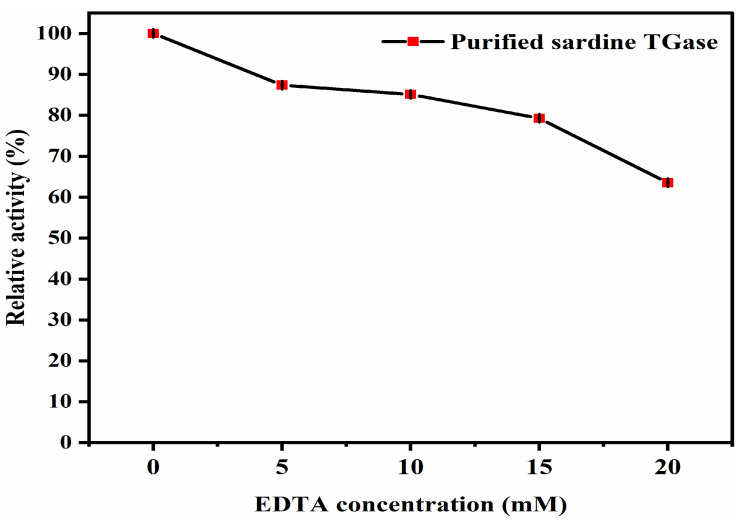
Effect of EDTA concentrations on the purified sardine TGase activity. The protein concentration used was 0.14 mg/mL. Bars represent the standard deviation (n = 3).

EDTA is the most common chemical compound used as a chelator worldwide. Chelators are molecules that are able to form stable complexes with a metal ion, maintaining it in solution while suppressing its chemical activity [37]. EDTA is a highly stable and powerful complexing agent of metals with a wide industrial use [38]. EDTA compounds are used in the food industry as sequestrants and stabilizing agents, improving color and flavor stability, or as a vehicle for iron fortification [37]. Thus, testing the effect of EDTA as an inhibitor of sardine TGase activity is of great interest. Figure 11 shows a progressive reduction in the TGase activity, with an increase in the EDTA concentration from 0 to 20 mM. TGase lost about 36.49% of its activity at 20 mM EDTA. TGase from tilapia, threadfin bream liver, common carp, and oil sardine was also inhibited by this chelating agent [4,12,32]. In this study, the partial inhibition was due to the fact that EDTA is known as a calcium- and sulfhydryl-chelating agent, which reacts with a thiol group. These results support the view that sardine TGase is absolutely dependent on calcium ions and contains a thiol group at the active site.

##### Effect of Metal Ions on Transglutaminase Activity

Metal ions have negative impacts on various industrial processes and on the formulation of different products. The presence of transition metal ions, such as those of iron, manganese, copper, and zinc, may trigger the chemical processes of corrosion, redox reactivity, polymerization inhibition, catalytic degradation, and changes in the coloring of products. Moreover, divalent ions like Ca^2+^, Mg^2+^, Mn^2+^, Zn^2+^, Fe^2+^, and Ba^2+^ form insoluble precipitates with carbonates, sulfates, and phosphates. In industrial processes, these metal ions may come from the raw materials, process water, equipment erosion and corrosion, or be added as a specific metal species. However, they may later suffer unwanted alterations due to oxidation, changes in the concentration and pH, or reactions with other ingredients during the process [38]. Additionally, metal ions have the capacity to be associated with proteins and can also form complexes with other molecules that are linked to enzymes acting as structural regulators or as electron acceptors or donors. These ions can either activate or inhibit the enzymatic activity by interacting with the amine or carboxylic acid groups of the amino acids [39]. For these reasons, the relative activity of TGase was investigated in the presence of several metal ions. The results (Table 3) showed that the enzyme activity was greatly affected by the addition of metal ions. The sardine TGase activity was activated by Mg^2+^ and Ba^2+^ to a greater extent but less than by Ca^2+^ at the same concentration (10 mM). The binding of Mg^2+^ and Ba^2+^ to the TGase molecule induced the exposure of the sulfhydryl active site, leading to half (55.2 ± 1.58%) and a quarter (24.91 ± 1.6%) of the relative activity, respectively. The results also showed that TGase was completely inhibited (0% of relative activity) when treated with Sr^2+^ and Zn^2+^ ions. Thus, the activation of sardine TGase by the tested ions increased in the following order: Sr^2+^ and Zn^2+^ < Ba^2+^ < Mg^2+^. These findings are in accordance with the study of Laksono et al. [7], where the TGase activity in daggertooth pike conger fish meat was activated the greatest (two-fold increase) by Mg^2+^ metal ions, reduced by Zn^2+^ and Cu^2+^, and inactivated by Fe^2+^ metal ions. Zhang et al. [8] also stated that the inhibition of Antarctic krill TGase by metal ions decreased in the following order: Cu^2+^ > Zn^2+^ > Ba^2+^ > Mn^2+^ > Mg^2+^. However, the TGase activity of tilapia was inhibited by Ba^2+^ and Mg^2+^, reduced by Sr^2+^ up to 55%, and completely inhibited by Cu^2+^ and Zn^2+^. It is well known that metal ions such as Zn^2+^ have a strong affinity for the thiol group of the cysteine residues, which represents a part of the enzyme’s active site and thus reduces or inhibits the activity of the enzyme completely. This result confirmed the idea that sardine TGase could have a thiol group at the active site. However, the amino acids in the TGase active site may interact with other metal ions, such as Mg^2+^ and Ba^2+^ [8,31]. These results indicated that Sr^2+^ can bind strongly to the amino acids at the active site of TGase and suggested that this metal ion could become a TGase inhibitor. From these results, it could be concluded that even though Mg^2+^ and Ba^2+^ activated the enzyme activity to a greater extent, their concentrations are too low to induce the significant conformational changes necessary for the catalytic reaction of TGase like the Ca^2+^ ions.

### 3.3. Effect of Added Sardine Transglutaminase on the Natural Actomyosin (NAM) Cross-Linking

#### 3.3.1. Protein Solubility

**Figure 12:**
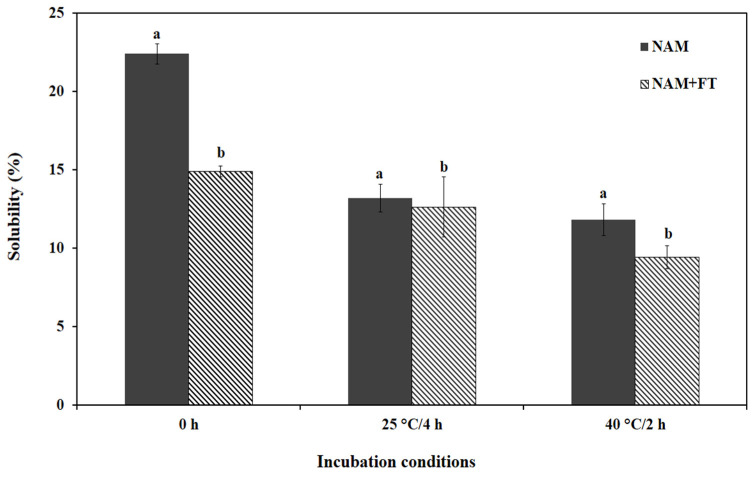
Protein solubility of incubated natural actomyosin samples. **NAM** natural actomyosin; **NAM+FT** natural actomyosin treated with fish transglutaminase; 25 °C/4 h incubation at 25 °C for 4 h; 40 °C/2 h incubation at 40 °C for 2 h. Bars represent the standard deviation (n = 3). Different letters within the same thermal treatment indicate significant differences (*p* < 0.05).

Protein solubility is an important index to evaluate protein aggregation–denaturation, which directly affects most of the functional properties of proteins, including gelation, emulsification, and foaming [40]. As shown in Figure 12, and for the different incubation conditions, the solubility of the samples treated with fish TGase (NAM+FT) presented a higher degree of precipitation and a significantly lower solubility (*p* < 0.05) than those of the samples not treated with sardine TGase. Similar findings regarding the increase in protein solubility in pork myofibrillar protein treated with MTGase in different pH conditions were reported by Hong and Xiong [41]. According to Ali et al. [42], the increase in protein solubility suggests that the substrates contain sites that are recognizable by the enzyme. The deamidation reaction promoted by the action of TGase increases the electrostatic repulsion between protein chains, leading to an increase in their solubility [5]. After incubation at either 25 or 40 °C, the solubility of different NAM samples decreased significantly (*p* < 0.05), indicating the establishment of intra- and inter-molecular interactions to form aggregates to a greater extent. It should be noted that NAM-FT exhibited a higher solubility after incubation at 40 °C/2 h. These results are in harmony with those found by Hemung et al. [25], who stated that the solubility of Pacific whiting NAM decreased after heat treatment at both 25 °C/4 h and 40 °C/2 h. During thermal treatment, hydrophobic interactions were responsible for the aggregate formation of NAM [25].

#### 3.3.2. Surface Hydrophobicity

**Figure 13:**
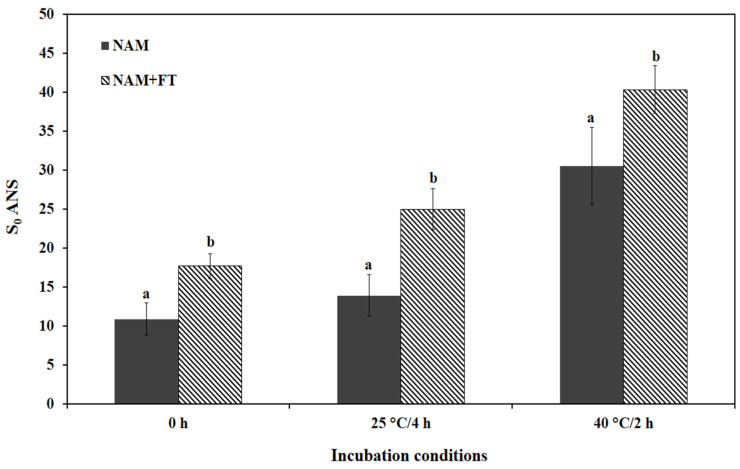
Surface hydrophobicity of incubated natural actomyosin samples. **NAM** natural actomyosin; **NAM+FT** natural actomyosin treated with fish transglutaminase; 25 °C/4 h incubation at 25 °C for 4 h; 40 °C/2 h incubation at 40 °C for 2 h. Bars represent the standard deviation (n = 3). Different letters within the same thermal treatment indicate significant differences (*p* < 0.05).

Surface hydrophobicity is an important parameter related to the modification of the protein structure, which affects the gelling, emulsifying, and foaming capacities [43]. Fluorescence intensity measurements indicated that the NAM+FT sample had a significant (*p* < 0.05) increase in the surface hydrophobicity as a function of transglutaminase addition (Figure 13). Increased surface hydrophobicity indicates conformational changes of the protein. When ε-amino groups of lysine residues in proteins act as acyl acceptors, intra- and intermolecular ε-(ɣ-glutamyl)-lysine (G-L) cross-links are formed. These isopeptide bonds create a stable protein network, which is important in the formation of gels and produces changes in the hydrophobicity of the protein surface [43]. After incubation at either 25 or 40 °C, the S_0_ ANS of the NAM+FT samples increased significantly (*p* < 0.05) compared with the control NAM, with the highest surface hydrophobicity recorded for NAM+FT incubated at 40 °C/2 h. Similar results were reported by Hemung et al. [25], who stated that the S_0_ ANS of Pacific whiting and threadfin bream NAMs increased after thermal treatment (40 °C/2 h and 25 °C/4 h).

## 4. Conclusions

The present study succeeded in obtaining a newly purified sardine (*Sardina pilchardus*) transglutaminase (TGase) after three purification steps, including ammonium sulphate precipitation, ion exchange (DEAE-Sepharose), and size exclusion (HiPrep Sephacryl S-300) chromatographies. Sardine TGase had the highest specific activity (357.14 U/mg protein) compared with the other marine fish species reported in previous studies. The endogenous TGase had an optimal temperature and pH of 40 °C and 8.0, respectively, and was stable for up to 20 min at 40 °C (relative activity of 100%). The sardine TGase was activated by CaCl_2_, confirming its dependency on calcium ions. Mg^2+^ and Ba^2+^ activated the enzyme activity to a greater extent; however, their concentrations are too low to induce the significant conformational changes necessary for the catalytic reaction of TGase compared with the Ca^2+^ ions. Furthermore, the enzyme activity was totally inhibited by Zn^2+^ and Sr^2+^ cations. When adding EDTA to the reaction mixture, TGase activity was markedly reduced, suggesting that the purified enzyme probably had a thiol group in its catalytic site. Moreover, TGase activity decreased with increasing NaCl concentrations up to 19.3% at 1.5 M due to conformational changes induced in the enzyme active site. Based on the surface hydrophobicity and solubility results, the cross-linking of NAM from sardine fish underwent greater conformational changes under setting conditions at either 25 °C for 4 h or 40 °C for 2 h, and such changes directly governed the extent to which protein cross-linking was catalyzed by TGase. These results suggest that sardine TGase exhibited unique and interesting characteristics compared with TGases from other marine species and sources. These distinct properties make this endogenous TGase a good alternative to be used in the food industry and pave the way for future studies on the effect of sardine TGase on cross-linking proteins in fish- and meat-based products. However, additional investigation is required to: (i) study the molecular characterization; (ii) improve the yield during extraction and purification; and (iii) optimize the standard TGase activity assay protocol conditions (concentration of TGase, use of Mg^2+^ ions as activator in addition to Ca^2+^, pH, temperature, time) to enable more efficient utilization of under-exploited sardine as a potential source of TGase for commercial scale use.

## Figures and Tables

**Table 1:** Purification steps of TGase from sardine (*Sardina pilchardus*) flesh.

Purification Step	Total Protein * (mg/mL)	Total Activity (U/mL)	Specific Activity (U/mg Protein)	Purification Fold	Yield (%)
Crude extract	862.29	1683.00	1.95	1.00	100.00
Dialyzed fraction	227.84	156.00	0.68	0.35	98.11
DEAE-Sepharose	0.84	136.08	162.16	83.15	87.23
Hiprep Sephacryl S-300	0.14	50.00	357.14	183.15	36.74

* From 250 g of sardine flesh.

**Table 2:** TGases activities and yields of various fish species.

Common Name	Species	Specific Activity (U/mg)	Yield (%)	References
Sardine	*Sardina pilchardus*	357.14	36.74	This study
Daggertooth pike conger	*Muraenesox cinerus*	3.65	17.14	Lakonso et al. [31]
Bigeye snapper	*Priacanthus hamrur*	47.44	29.54	Binsi and Shamasundar [4]
Oil sardine	*Sardinella longiceps*	66.35	24.85	
Tilapia	*Oreochromis mossambicus*	69.14	22.66	
Common carp	*Cyprinus carpio*	49.33	33.01	
Tropical tilapia	*Oreochromis niloticus*	197.00	12.90	Warratao and Yongsawatdigul [12]
Carp	*Cyprinus carpio*	8.60	24.00	Nozawa et al. [14]
Rainbow trout	*Oncorhynchus mykiss*	1.17	21.00	
Atka mackerel	*Pleurogrammus azonus*	1.10	2.00	

**Table 3:** Effect of metal ions on sardine TGase activity.

Reagent	Concentration (mM)	Relative Activity (%)
Control: CaCl_2_	10	100
Ba^2+^	10	24.91 ± 1.6
Mg^2+^	10	55.2 ± 1.58
Sr^2+^	10	0
Zn^2+^	10	0

All results are expressed as the means ± standard deviation.

## Data Availability

The data presented in this study are available upon request from the corresponding author.
